# Scalp-Sparing Radiation With Concurrent Temozolomide and Tumor Treating Fields (SPARE) for Patients With Newly Diagnosed Glioblastoma

**DOI:** 10.3389/fonc.2022.896246

**Published:** 2022-04-29

**Authors:** Ryan Miller, Andrew Song, Ayesha Ali, Muneeb Niazi, Voichita Bar-Ad, Nina Martinez, Jon Glass, Iyad Alnahhas, David Andrews, Kevin Judy, James Evans, Christopher Farrell, Maria Werner-Wasik, Inna Chervoneva, Michele Ly, Joshua Palmer, Haisong Liu, Wenyin Shi

**Affiliations:** ^1^ Department of Radiation Oncology, Thomas Jefferson University, Philadelphia, PA, United States; ^2^ Department of Neuro-Oncology, Thomas Jefferson University, Philadelphia, PA, United States; ^3^ Department of Pharmacology and Experimental Therapeutics, Thomas Jefferson University, Philadelphia, PA, United States; ^4^ Sidney Kimmel Medical College, Thomas Jefferson University, Philadelphia, PA, United States; ^5^ Department of Radiation Oncology, The Ohio State University, Columbus, OH, United States

**Keywords:** TTFields, glioblastoma, scalp-sparing radiation, concurrent therapy, radiotherapy

## Abstract

**Introduction:**

Standard-of-care treatment for patients with newly diagnosed glioblastoma (GBM) after surgery or biopsy includes concurrent chemoradiation followed by maintenance temozolomide (TMZ) with tumor treating fields (TTFields). Preclinical studies suggest TTFields and radiotherapy work synergistically. We report the results of our trial evaluating the safety of TTFields used concurrently with chemoradiation.

**Methods:**

This is a single-arm pilot study (clinicaltrials.gov Identifier: NCT03477110). Adult patients (age ≥ 18 years) with newly diagnosed glioblastoma and a Karnofsky performance score (KPS) of ≥ 60 were eligible. All patients received concurrent scalp-sparing radiation (60 Gy in 30 fractions) with TMZ (75 mg/m^2^ daily) and TTFields (200 kHz). Maintenance therapy included TMZ and continuation of TTFields. Scalp-sparing radiation treatment was used to reduce radiation dermatitis. Radiation treatment was delivered through the TTFields arrays. The primary endpoint was safety and toxicity of tri-modality treatment within 30 days of completion of chemoradiation treatment.

**Results:**

There were 30 patients enrolled, including 20 (66.7%) men and 10 (33.3%) women, with a median age of 58 years (range 19 to 77 years). Median KPS was 90 (range 70 to 100). A total of 12 (40%) patients received a gross total resection and 18 (60%) patients had a subtotal resection. A total of 12 (40%) patients had multifocal disease at presentation. There were 20 (66.7%) patients who had unmethylated O(6)-methylguanine-DNA-methyltransferase (MGMT) promotor status and 10 (33.3%) patients who had methylated MGMT promoter status. Median follow-up was 15.2 months (range 1.7 to 23.6 months). Skin adverse events were noted in 83.3% of patients, however, these were limited to Grade 1 or 2 events, which resolved spontaneously or with topical medications. The primary end point was met; no TTFields discontinuation occurred during the evaluation period due to high grade scalp toxicity. A total of 27 (90%) patients had progression, with a median progression-free survival (PFS) of 9.3 months (95% confidence interval (CI): 8.5-11.6 months). The 1-year progression-free survival was 23% (95% CI: 12%-45%). The median overall survival (OS) was 15.8 months (95% CI: 12.5 months-infinity). The 1-year overall survival was 66% (95% CI: 51%-86%).

**Conclusions:**

Concurrent TTFields with scalp-sparing chemoradiation is a feasible and well-tolerated treatment option with limited toxicity. A phase 3, randomized clinical trial (EF-32, clinicaltrials.gov Identifier: NCT04471844) investigating the clinical benefit of concurrent TTFields with chemoradiation treatment is currently enrolling.

**Clinical Trial Registration:**

Clinicaltrials.gov, identifier NCT03477110.

## Introduction

Concurrent chemoradiation and maintenance temozolomide (TMZ) with tumor treating fields (TTFields) is a category 1 recommendation for patients with newly diagnosed glioblastoma (GBM) (1). Addition of TMZ concurrently with radiation, and as maintenance therapy, has shown significant improvements in both progression-free survival (PFS) and overall survival (OS) (2, 3). Furthermore, the addition of TTFields to maintenance TMZ chemotherapy has led to improvements in both outcomes (4, 5). TTFields uses low intensity, intermediate frequency (200 kHz) alternating electric fields to arrest cell proliferation and disrupt cancer cell replication (6, 7). Preclinical models have demonstrated that TTFields causes mitotic arrest and apoptosis by disrupting mitotic spindle formation during metaphase; furthermore, it causes dielectrophoretic movement of polar molecules during cytokinesis (6–9). TTFields has not been associated with an increase in systemic adverse events compared with TMZ alone; however, previous studies have shown an increase in mild-to-moderate skin irritation (~52%) under the transducer arrays (5).

The concept of introducing TTFields not only as maintenance therapy, but concurrently with chemoradiation is supported by prior clinical work. A pilot study which combined TTFields treatment concurrently with chemoradiation in newly diagnosed GBM demonstrated a median PFS of 8.9 months, which is in comparison to a median PFS of 6.9 months and 6.7 months in the European Organisation for Research and Treatment of Cancer/National Cancer Institute of Canada Clinical Trials Group (EORTC/NCIC) and EF-14 studies, respectively (3, 5, 10). In addition to introducing TTFields earlier, it has been demonstrated that compliance and longer use lead to better outcomes, with > 90% usage leading to a median survival of 24.9 months on an EF-14 subgroup analysis (11). Administering TTFields concurrently with chemoradiation is also supported by preclinical studies in glioma cell cultures (12, 13). These studies have shown that administration of TTFields with radiation demonstrates synergy, possibly by inhibition of DNA damage repair (12). Cell apoptosis, DNA damage, and mitotic abnormalities were increased when TTFields were combined with ionizing radiation (13).

Currently, the only clinical study reported in the literature which combined TTFields with chemoradiation included 10 patients, for which the electrode array was removed during radiation treatment (10). On this study, 80% of patients experienced Grade 1-2 TTFields-related skin toxicity; no other TTFields-related toxicities were reported and there was no increase in chemoradiation-related toxicity. At our institution, a previous study was published on a cranial radiation phantom model with transducer arrays in place (14). This study demonstrated that there was minimal impact on deep dose measurements with a mean reduction of planning treatment volume (PTV) dose by 0.5 to 1%. However, there was an increase in measured surface dose by a mean ratio of 2.2 for a volumetric modulated arc therapy (VMAT) plan and the scalp dose was increased by a mean of 0.5 to 1.0 Gy. Due to the known skin toxicity associated with TTFields as discussed above, a scalp-sparing radiation treatment volume adjustment was used for our current study.

Previously, a 10-patient initial experience by our institution was published and showed a skin toxicity of 80% and was limited to Grade 1-2 events which resolved spontaneously or responded to topical medications (15). We report the final results with 30 patients evaluating toxicity and tolerability of Scalp Preservation Chemoradiation Plus Alternating Electric Tumor Treating Fields (SPARE) with delivery of radiation through the transducer arrays, followed by maintenance TMZ and TTFields.

## Materials and Methods

This study was approved by the Institutional Review Board (IRB) at Thomas Jefferson University in Philadelphia, PA.

### Study Population

Eligible patients included those adults, age ≥ 18 years, with a Karnofsky performance score (KPS) ≥ 60, with a pathology-confirmed diagnosis of World Health Organization (WHO) Grade IV glioma. Patients had to have adequate hematologic, hepatic, and renal function. Patients with infratentorial disease, implanted pacemaker, defibrillator, deep brain stimulator, skull defects, known hypersensitivity to conductive hydrogels, non-healing surgical incision or wounds on the scalp, and prior radiation and/or TMZ were excluded.

### Study Design

This single-arm pilot trial was conducted at Thomas Jefferson University in Philadelphia, PA with the intent of evaluating the safety and toxicity of combination chemoradiation with TTFields for newly diagnosed GBM. Patients were treated with concurrent scalp-sparing radiation (60 Gy in 30 fractions), TMZ (75 mg/m^2^), and TTFields (200 kHz). Patients had to have recovered from the effects of surgery per the treating physician’s judgment; there was a minimum of 21 days from the day of surgery to the initiation of protocol treatment; for core or needle biopsy, there was a minimum of 14 days from the day of biopsy to the initiation of protocol treatment. Concurrent treatment started ≤ 7 weeks from the time of surgery or biopsy.

Following the concurrent phase, patients continued TTFields without interruption as tolerated. After 4 weeks (28 days, with up to additional 7 days), maintenance TMZ was started for 12 cycles on Days 1-5 every 28 days, unless there was disease progression, intolerable toxicity, voluntary withdrawal, or death.

### Primary and Secondary Endpoints

The primary endpoint of the study was the discontinuation rate of TTFields due to skin toxicity during the concurrent chemoradiation phase and up to 30 days after completion of the concurrent phase. Discontinuation events were defined as discontinuation of TTFields for > 7 days due to a skin toxicity of Grade 3 or higher.

Secondary endpoints included median PFS, defined as the time from the start of radiation treatment to first disease progression or death, and median OS, which was measured from the start of radiation treatment to death. An additional secondary endpoint included quality of life evaluation for patients treated with concurrent chemoradiation and TTFields with maintenance TMZ and TTFields.

### Pathology and Molecular Testing

Tumor O(6)-methylguanine-DNA-methyltransferase (MGMT) methylation status and isocitrate dehydrogenase (IDH) mutation status were tested on tumor specimens at the Department of Pathology at Thomas Jefferson University in Philadelphia, PA.

### Radiation Treatment

At the time of simulation, patients were immobilized in the supine position using a Brainlab thermoplastic mask (Brainlab, Munich, Germany) using custom latex-free open cell styrene butadiene rubber (SBR) foam (Jaybird & Mais, Lawrence, MA) cutouts to accommodate patients’ TTFields transducer arrays. All patients underwent treatment planning computed tomography (CT) imaging, which was fused with post-operative magnetic resonance (MRI) imaging for target delineation and identifying organs at risk (OARs).

Regarding contours, the protocol initially defined target volumes following EORTC guidelines (16). These guidelines define the gross tumor volume (GTV) as the T1 post-contrast enhancing lesion and surgical cavity. The clinical target volume (CTV) was a 2- cm expansion of the GTV with adjustments made around natural barriers to tumor growth, such as the skull, falx, or tentorium. The planning target volume (PTV) was a uniform 3-mm expansion of the CTV. Radiation was prescribed as 60 Gy in 30 fractions with 95% of the PTV receiving 100% of the prescribed dose. The protocol was revised to allow Radiation Therapy Oncology Group (RTOG) target guidelines as well to accommodate provider preference (16). In their guidelines, GTV1 was defined as the T1 post-contrast enhancing lesion and surgical cavity, as well as any FLAIR abnormality. CTV1 was a 2-cm expansion on the GTV1 with a reduction around natural barriers to tumor growth as listed above. PTV1 was a 3-mm uniform expansion of the CTV1. Radiation was prescribed as 46 Gy in 23 fractions to PTV1. GTV2 included the T1 post-contrast enhancing lesion and surgical cavity only. CTV2 was a 2-cm expansion of the GTV2 with a reduction around natural barriers to tumor growth as listed above. PTV2 was a 3-mm uniform expansion of the CTV2. PTV2 was prescribed to 14 Gy in 7 fractions, with a total accumulated dose of 60 Gy in 30 fractions.

The scalp was used as an avoidance structure for planning and was defined as 5-mm thickness from the skin surface above the level of the foramen magnum. The following dose constraints were used: mean < 20 Gy, D20cc < 50 Gy, and D30cc < 40 Gy (14). PTV coverage was prioritized over scalp dose constraints when necessary. The Volumetric Modulated Arc Therapy (VMAT) technique was utilized. All radiation treatment planning was done with Eclipse (Varian, Palo Alto, CA), and all patients were treated with TrueBeam STx (Varian, Palo Alto, CA) with daily ExacTrac (Brainlab, Munich, Germany) image guidance.

At the time of treatment, radiation was delivered through the TTFields arrays. The power supply was disconnected before treatment and left outside the radiation vault; after the radiation treatment, the device was reconnected and resumed promptly.

### Tumor Treating Fields

TTFields started concurrently with the first day of radiation treatment (with up to 1 week acceptable). TTFields was administered continuously with a planned ≥ 18 h per day duration of usage. If a patient required an intervention prohibiting the use of TTFields, such as a surgery for recurrence, then TTFields could be discontinued for ≤ 60 days before resuming. Monthly device logs were obtained for all patients on trial, including average daily use (ADU). Non-adherence to TTFields was defined as < 75% ADU per monthly device logs absent of any medical indication necessitating an interruption in treatment, such as skin reaction or ulceration.

The scalp skin below the electrode was inspected by the physician and/or patient during transducer array placement. The electrode location was shifted between two alternate sites at every electrode gel change (17).

### Response Assessment

Treatment response and disease progression were monitored with serial brain MRI imaging as per the Updated Response Assessment Criteria for High-Grade Gliomas: Response Assessment in Neuro-Oncology Work Group (RANO criteria) (18).

### Toxicity

The Common Terminology Criteria for Adverse Events (CTCAE) 5.0 was used to grade toxicity (19). If there was localized skin toxicity, including any breakdown or infection which required more than 3 days of treatment interruption, this was reported as an adverse event. If a patient developed skin toxicity prohibiting continued use of TTFields during the concurrent phase of treatment, TTFields would be discontinued while chemoradiation treatment was continued.

### Mental Status and Quality of Life Assessment

A Mini-Mental State Examination (MMSE) was administered to patients at baseline before starting the concurrent phase of treatment, during the concurrent phase of treatment, and during the maintenance phase of treatment. In addition, patients were given the EORTC Core Quality of Life questionnaire (QLQ-C30 version 3) and a brain cancer-specific health-related quality of life questionnaire (QLQ-BN20) to complete at these same timepoints. The QLQ-C30 version 3 questionnaire includes 30 questions which assess quality of life and is divided into several domains including global health status, physical functioning, role functioning, emotional functioning, cognitive functioning, social functioning, and general symptoms. QLQ-BN20 contains 20 questions which are more specific to brain cancer patients and includes domains such as future uncertainty, visual symptoms, motor dysfunction, communication deficit, and additional symptoms often experienced by these patients.

### Statistical Analysis

The TTFields discontinuation rate during the concurrent chemoradiation phase and within 30 days of completion due to skin toxicity was estimated with the corresponding 95% exact binomial confidence interval (CI). PFS and OS were evaluated using Kaplan-Meier estimates for the entire cohort and by methylation status. The estimates for PFS and OS were based on 30 patients. The difference in PFS or OS by methylation status was tested using the log-rank test. All statistical analyses were performed using R 4.0.4. Descriptive analysis was performed on acute toxicity data, as well as TTFields duration of usage and quality of life data.

## Results

### Study Participants

A total of 30 patients were enrolled in the trial (**Table 1**). There were 20 (66.7%) men and 10 (33.3%) women. The median age was 58 years (range 19-77 years). The median KPS was 90 (range 70-100). There were 20 (66.7%) patients who had unmethylated MGMT promotor status and 10 (33.3%) patients who had methylated promoter status. A total of 12 (40%) patients received a gross total resection and 18 (60%) patients had a subtotal resection. The median time from surgery to radiation was 34 days (range 26-49 days). The median follow-up was 15.2 months (range 1.7 to 23.6 months).

**Table 1:** Patient demographics.

Baseline characteristic	N (%)
**Gender**	
Men	20 (66.7%)
Women	10 (33.3%)
**Age**	
Median (range)	58 (19-77)
**Karnofsky performance score**	
Median (range)	90 (70-100)
**Extent of resection**	
GTR	12 (40%)
STR/biopsy	18 (60%)
**MGMT status**	
Methylated	10 (33.3%)
Unmethylated	20 (66.7%)
**Multifocal disease**	
Yes	12 (40%)
No	18 (60%)
**T-RT**	
Median (range)	34 (26-49)

GTR, gross total resection, STR, subtotal resection, MGMT, O(6)-methylguanine-DNA-methyltransferase, T-RT, time from surgery to radiation start in days.

### Treatment Delivery and Duration of Usage

The median scalp dose volume was 455 cc (range 352.3-682.7 cc). Scalp dose constraints were achieved for all patients in the trial. The mean dose median was 8.3 Gy (range 4.3-14.7 Gy), the D20 cc median was 25.9 Gy (range 17.7-42.8 Gy), and the D30 cc median was 23.5 Gy (range 14.8-35.4 Gy). The max dose median was 51.6 Gy (range 33.4-65.7 Gy).

Regarding the primary endpoint, the TTFields discontinuation rate during the concurrent phase and within 30 days of completion of the concurrent phase due to skin toxicity was 0% (95% CI: 0-11.6%). Regarding TTFields compliance, the median usage was 10.7 months (range 1.6-21.7 months) from the time of initiation. The ADU for patients during the concurrent phase had a median of 82.6% (range 9-92.5%). The ADU for patients during the maintenance phase had a median of 74.6% (range 0-91%).

### Toxicity

Regarding toxicity, no Grade 3 or higher toxicity during the concurrent or maintenance phase was observed on trial. Grade 1 events reported during the concurrent phase and up to 30 days after completion of the concurrent phase included 22 (73.3%) patients with skin toxicity (dermatitis, erythema, folliculitis), 9 (30%) with fatigue, 1 (3.3%) with cognitive impairment, 11 (36.7%) with pruritus, 3 (10%) with headache, 2 (6.7%) with dizziness, and 4 (13.3%) with nausea (**Table 2**). Grade 2 events reported during the concurrent phase and up to 30 days after completion of the concurrent phase included 3 (10%) patients with skin toxicity and 1 (3.3%) with headache (**Table 2**). No radiation treatment interruption occurred due to TTFields-related toxicity for the 30 patients on trial.

**Table 2:** Adverse events deemed possible or greater relatedness to therapy.

Adverse event	Grade 1, N (%)	Grade 2, N (%)
Dermatitis^a^	22 (73.3%)	3 (10%)
Pruritus	11 (36.7%)	
Fatigue	9 (30%)	
Headache	3 (10%)	1 (3.3%)
Nausea	4 (13.3%)	
Dizziness	2 (6.7%)	
Cognitive impairment^b^	1 (3.3%)	

No related adverse events ≥ Grade 3 during either concurrent or maintenance phases.

a Dermatitis included scalp irritation, dry skin, folliculitis, erythema, color change, or rash.

b Cognitive impairment included concentration change, memory change, or confusion.

### Progression-Free and Overall Survival

**Figure 1:**
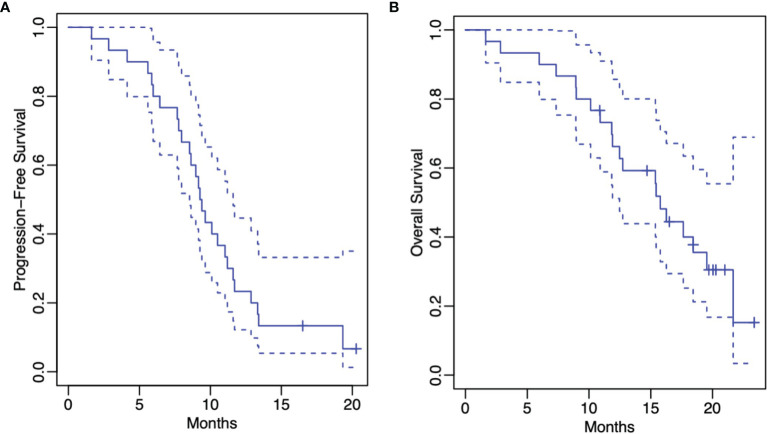
Kaplan-Meier estimates of progression-free survival **(A)** and overall survival **(B)** (both represented by solid lines). The dashed lines represent the corresponding 95% confidence pointwise confidence intervals.

**Figure 2:**
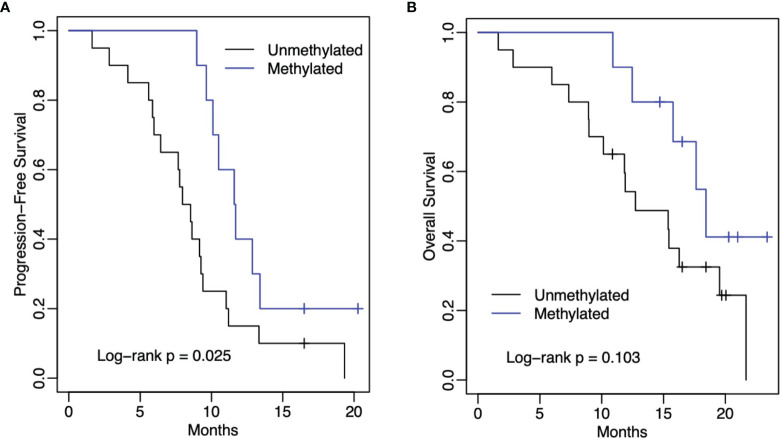
Kaplan-Meier estimates of progression-free survival **(A)** and overall survival **(B)** by methylation status. In both figure panels, the black line represents unmethylated MGMT promoter patients and the blue line represents methylated MGMT promoter patients.

At the time of this writing, 27 (90%) patients had progression. The median PFS for the entire cohort was 9.3 months (95% CI: 8.5-11.6 months) (**Figure 1**). The 1-year progression-free survival was 23% (95% CI: 12%-45%). The median PFS was 8.3 months for those patients without MGMT promotor methylation status (95% CI: 6.4 months-infinity) (**Figure 2**). The median PFS was 11.7 months for those patients with MGMT promotor methylation status (95% CI: 10.1 months-infinity) (**Figure 2**). A total of 4 (13.3%) patients were compliant with concurrent TTFields for > 90% ADU, and among these patients, the median PFS was 10.1 months (95% CI: 6.6-13.2 months).

The median OS for the entire cohort was 15.8 months (95% CI: 12.5 months-infinity) (**Figure 1**). The 1-year overall survival was 66% (95% CI: 51%-86%). The median OS was 12.7 months for those patients without MGMT methylation (95% CI: 10.1 months-infinity) (**Figure 2**). The median OS was 18.4 months for those patients with MGMT methylation (95% CI: 15.8 months-infinity) (**Figure 2**). Among the 4 patients compliant with concurrent TTFields for > 90% ADU, the median OS could not be reached.

### Mental Status and Quality of Life

The median MMSE score at baseline was 30 (range 1-30). The median MMSE during the concurrent phase of treatment was 29 (range 1-30). In comparison to baseline MMSE examination, the median change in score during the concurrent phase was 0 (range -7 to +5). Among the 6 patients with a decline in MMSE during the concurrent phase, 2 returned to baseline, 3 did not have a maintenance MMSE conducted, and 1 patient continued to decline during the maintenance phase.

**Figure 3:**
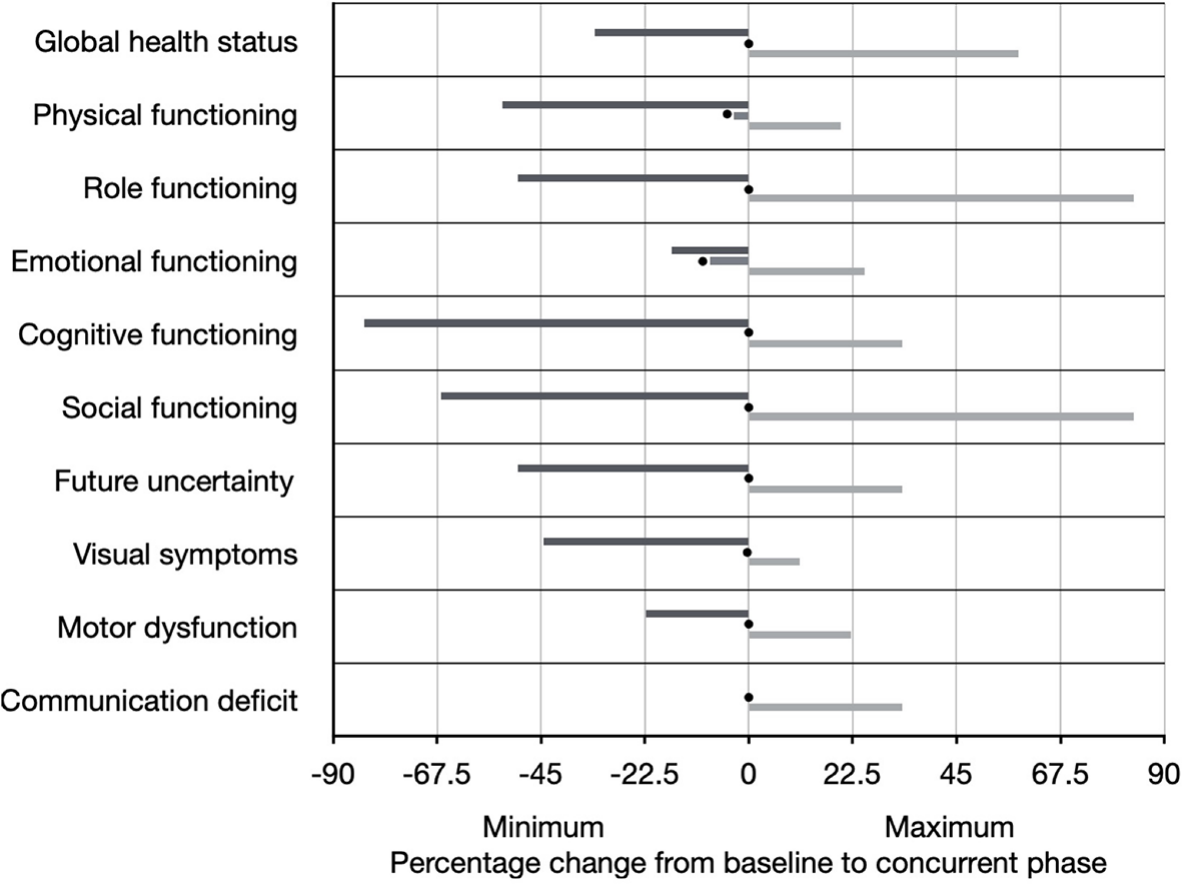
Quality of life as measured by the EORTC Core Quality of Life questionnaire (QLQ-C30 version 3) and brain cancer-specific health-related quality of life questionnaire (QLQ-BN20). Individual scales are shown on the Y-axis. The percentage change from baseline questionnaire administration to concurrent phase questionnaire administration (at approximately Week 3) is shown on the X-axis, including the range (black line represents minimum and gray line represents maximum), as well as the median (represented by black circle).

The median change in score between baseline and the concurrent phase for global health status was 0% (range -33.3% to +58.3%), for physical functioning was -3.3% (range -53.3% to +20%), for role functioning was 0% (range -50% to +83.3%), for emotional functioning was -8.3% (-16.7% to +25%), for cognitive functioning was 0% (-83.3% to +33.3%), and for social functioning was 0% (-66.7% to +83.3%) (**Figure 3**). The median change in score between baseline and the concurrent phase for future uncertainty was 0% (range -50% to +33.3%), for visual symptoms was 0% (range -44.4% to +11.1%), for motor dysfunction was 0% (range -22.2% to +22.2%), and for communication deficit was 0% (range 0% to +33.3%) (**Figure 3**).

## Discussion

Our trial is one of the first to combine TTFields concurrently with chemoradiation with the radiation treatment delivered through the TTFields arrays. No Grade 3 or higher adverse events were noted, and no patient discontinued TTFields due to skin toxicity. Grade 1 skin toxicity was 73.3% and Grade 2 skin toxicity was 10%, but these resolved spontaneously or responded to topical medications. No patient had an interruption in their radiation treatment course as a result of TTFields-related toxicity, and there was negligible change in mental status and quality of life between baseline and the concurrent treatment phase.

The other clinical study reported in the literature which combined TTFields with chemoradiation included 10 patients, for which the electrode array was removed during radiation treatment (10). On their study, 80% of patients experienced Grade 1-2 TTFields-related skin toxicity, similar to our results. They found a median PFS from enrollment to be 8.9 months. In contrast, EF-14 demonstrated a PFS of 6.7 months when TTFields was given with maintenance TMZ only (5). In our cohort, we found a median PFS of 9.3 months when TTFields was given concurrently with chemoradiation and with maintenance TMZ, which suggests a potential improvement in comparison to previously reported studies (3, 5, 10, 20, 21). A planned multi-institutional study is investigating a similar strategy of concurrent TTFields with chemoradiation and has a separate arm planned for elderly patients with reduced KPS who are receiving hypofractionated radiation (22). In addition, EF-32, or the TRIDENT trial, is a phase 3, randomized study currently enrolling that will further investigate introduction of TTFields concurrently with chemoradiation on clinical outcomes (23).

Patients with methylated MGMT promotor status in our study had a median PFS of 11.7 months and patients with unmethylated MGMT promotor status had a median PFS of 8.3 months. A recent systematic review and meta-analysis reported the median PFS for methylated patients treated with chemoradiation alone to be 9.51 months and for unmethylated patients to be 4.99 months (24). Taken together, it is promising that the addition of TTFields concurrently with chemoradiation leads to benefit regarding disease progression for both MGMT methylated and unmethylated patients.

Overall survival for the cohort was 15.8 months, which was less than in EF-14 (5). This could be due to the number of patients who underwent a subtotal resection (60%), as well as the number of patients who had multifocal disease at diagnosis (40%) including 1 (3.3%) patient with gliomatosis, a presentation known to lead to worse prognosis (25). Furthermore, 2 (6.7%) patient deaths early in the trial were attributed to non-GBM-related causes (sepsis and pulmonary embolism). Last, the population in our study included patients with early disease progression, which was excluded in EF-14 (5). Patients with progression received salvage therapy and, thus, we acknowledge that OS is not an optimal indicator to evaluate the efficacy of a first line treatment. Nevertheless, the data presented is hypothesis generating and provides support regarding the safety and feasibility of concurrent TTFields. PFS and OS will be further investigated in a phase 3 trial (23).

Regarding salvage treatments for patients with recurrent disease, 16 (53.3%) continued TTFields, 15 (50%) received Bevacizumab, 11 (36.7%) underwent re-resection, 9 (30%) received further radiation, 9 (30%) received lomustine, and 4 (13.3%) received immunotherapy. Four (13.3%) patients experienced significant early progression of disease and elected to pursue hospice measures rather than additional treatments.

This study is a pilot and had a small sample size of 30 patients. In addition, overall survival data is still maturing for the patients alive at last follow up. Nevertheless, the patient population enrolled demonstrated adherence to the treatment protocol, both during the concurrent and maintenance phases as shown by the ADU results. There are barriers to accepting TTFields from a patient experience, with approximately 65% declining this treatment due to personal reasons or lack of social support when offered in both the primary and recurrent settings (26). By allowing patients on this trial to undergo radiation treatment with their TTField arrays in place has the potential to reduce some of the possible reluctance that comes with electrode reapplication. In the maintenance setting, 75% of patients were adherent with TTFields in EF-14, similar to our study (5). This demonstrates that adding TTFields concurrently with chemoradiation does not negatively affect adherence during subsequent maintenance therapy. In addition, quality of life scores did not significantly decline during the concurrent phase and for most patients when there was a reduction, they had a return to baseline during the maintenance phase.

Our precautions to create a separate volume for the scalp and follow the dose constraints specified in the methods limited TTFields-related skin toxicity to either Grade 1 or 2 only, and as a result, no patient had a break in their treatment course. In summary, we conclude that concurrent TTFields and scalp-sparing chemoradiation is a safe and feasible option with limited toxicity. This trial provides feasibility data for further investigation. A phase 3, randomized study (EF-32, clinicaltrials.gov Identifier: NCT04471844) is currently enrolling and is investigating the clinical benefit of concurrent TTFields with chemoradiation treatment.

## Data Availability Statement

The raw data supporting the conclusions of this article will be made available by the authors, without undue reservation.

## Ethics Statement

The studies involving human participants were reviewed and approved by Institutional Review Board at Thomas Jefferson University. The patients/participants provided their written informed consent to participate in this study.

## Author Contributions

Concept and design: WS; acquisition, analysis, or interpretation of data, drafting and review of the manuscript: all authors; statistical analysis: IC. All authors contributed to the article and approved the submitted version.

## Funding

Novocure with Grant No. 19-0065 provided funding for the trial. The funder was not involved in the study design, collection, analysis, interpretation of data, the writing of this article or the decision to submit it for publication.

## Conflict of Interest

WS: Consulting for Brainlab, Varian, and Novocure; research funding for clinical trial from Novocure and Regeneron.

The remaining authors declare that the research was conducted in the absence of any commercial or financial relationships that could be construed as a potential conflict of interest.

## Publisher’s Note

All claims expressed in this article are solely those of the authors and do not necessarily represent those of their affiliated organizations, or those of the publisher, the editors and the reviewers. Any product that may be evaluated in this article, or claim that may be made by its manufacturer, is not guaranteed or endorsed by the publisher.
